# Schools for Tubercular Children


**Published:** 1912-01-27

**Authors:** 


### THE SCHOOL CLINIC.*

### Schools for Tubercular Children.

THE notification of tuberculosis may raise questions connected with the attendance of children at school. A delicate child discovered in the house of a phthisical adult patient may be considered to be possibly suffering from tuberculosis and excluded on that ground from an ordinary day-school. The school for tubercular children which is connected with the Paddington Dispensary for Tuberculosis appears to have been established upon some such principle. This school may be described as an open-air school for children considered to be suffering from tuberculosis, in which the temperatures of the children are taken twice daily and an amount of rest prescribed which varies according to the temperature. The children whose temperatures rise to 100° Fahr. in the afternoon lie down all the time that they are at school. Those whose temperatures rise above 99° but do not reach 100° have less rest, but are not allowed to drill or to indulge in games entailing physical exercise. Upon children in whom the temperature does not reach 99° no restrictions are placed.

It is evident from the adoption of this system that a slight rise of temperature is considered to afford evidence of the presence of active tuberculosis. The question naturally occurs to the mind whether it may not be unreasonable to lay great stress upon a slight rise of temperature. There are many simple facts in medicine of which little is known, and many observations would need to be required upon the temperature of delicate and anæmic children before it could be stated that minor degrees of elevation indicate the presence of active tuberculosis. Whether, however, those responsible for the treatment of these children are or are not right in considering the temperature to be of great importance, it is wise to assume that delicate children coming from homes in which some relative suffering from tuberculosis is or has been present require especial care. Although the writer of this article holds the view that chronic tuberculosis of the lungs in children is very uncommon, a very large number of children have received tubercular infection in some part of the body, most commonly in the lymphatic glands connected with the lungs. Children coming from homes in which chronic tuberculosis has been present may easily acquire the infection at least in some slight degree. Not only will this be the case, but the children of tubercular parents, as is well known, have less power of resistance to inroads of the disease than children of parents not so affected. In these children, therefore, a quiescent focus of tuberculosis is more likely to light up into active disease.

While, however, such children require care, whether open air and rest are the main requirements as a protection against disease is a matter for consideration. In this school at Paddington no food is supplied. The children bring their own food which is warmed for them, or they go home to dinner. If rest is a very essential feature, it appears to be unreasonable that some children should be obliged to walk a mile home and a mile back—two miles in all—for the midday meal. Apart, however, from this, there can be no question that underfed children already handicapped by the presence of tuberculosis in the family cannot be expected to fight against tuberculosis with the same hope of success as children more happily placed as regards food. It is necessary to remember also that it may be the bread-winner who has been removed by disease. Possibly the organisation looks after special cases and provides material aid in the homes. Allowing, however, that such may be the case, most of us will probably consider that if children are taken from some surroundings and placed in others ostensibly for their own good no reasonable means should be omitted for providing this good. In other open-air schools a meal is provided for twopence, paid by the parents. This does not pay for the meal, but far more can be provided by clever management for twopence than might be expected. In this instance apparently most of the parents have been anxious to pay the twopence, and it is to be hoped that if the school is continued that the Committee will see their way to follow the system adopted in other schools in this matter.

A few other details may be mentioned. Felt-lined wooden clogs are being supplied for the children to use on going to and returning from school during the winter. The employment of these clogs should aid to prevent what is familiarly known as "catching cold." Of recent years there has been a tendency in great measure to disregard some old-fashioned notions. Some people, however, are peculiarly susceptible to evil effects following getting the feet wet. With them nasal or bronchial catarrh is almost invariably the result. Every child is weighed once a week, one class being weighed every day. The weight is recorded upon a chart. The taking of the temperatures and the weighing of the children are part of the duties of a trained nurse, who also, however, visits many of the homes. In conclusion, it should be mentioned that there are 116 children on the books and an average daily attendance of about 100. In virtually all cases one member of the family is said to have died of phthisis or is at the present time suffering from the disease. The school is established in a large empty house with a moderate sized garden. In London it must be difficult to find accommodation suitable for the establishing of a school for tubercular children. Although such a building as has been procured may serve a purpose from the point of view of an experiment, it is pervaded by a certain degree of atmosphere of makeshift which leaves in the mind the idea that if such schools are to be considered necessary, that it is desirable something more suited to the comfort and treatment of the children and the requirements of teaching should be provided.

* Previous articles in this series appeared in THE HOSPITAL of October 14, October 28, November 11, November 25, December 9, 1911, and January 6.

